# On the Present State of Medicine in Denmark

**Published:** 1836-07

**Authors:** C. Otto

**Affiliations:** Professor of Medicine in the University of Copenhagen


### THE PRESENT STATE OF MEDICINE IN DENMARK,

By C. Otto, M.D., Professor of Medicine in the University of Copenhagen.

### FIRST REPORT.

### ON THE MEDICAL INSTITUTIONS AND THE MEDICAL PROFESSION.

There are two public establishments for medical and surgical instruction in Denmark; the one the *Medical Faculty* at the university, the other the *College of Surgery.*

### A. THE MEDICAL INSTITUTION AT THE UNIVERSITY.

Denmark possesses two universities; one for the German provinces, at Kiel in Holstein; another for the Danish provinces in Copenhagen, on the Island of Zealand. The university of Kiel is, in almost all respects as to plan, the period of study, &c. like those in Germany; but that of Copenhagen, although in the beginning principally modelled after its sisters in Germany, differs at present in many respects from them.

### I. THE UNIVERSITY OF COPENHAGEN.

This was founded in the year 1479. The number of the professors, who are divided into the four usual Faculties, amounts at present to thirty-eight; but there are, besides these, one or two private teachers in every one of the Faculties. Some (the oldest) of the Professors are *Ordinary*, others (the younger) *Extraordinary.* The Ordinary Professors have a higher rank, have a seat in the *Consistorium* (the academical senate,) and a better salary. Every year a new rector of the University is chosen, and out of every faculty alternately, but only from among the ordinary professors. The rector has during his office a very high rank, is addressed " Your Magnificency," and gets a salary of about four hundred Danish dollars. The income granted to the professors is partly derived from domains and farms given to the University at its foundation, partly from rents of churches, and partly from royal and private bequests.

The buildings of the university were for the most part burnt in the bombardment by the English in 1807, and were not rebuilt until the last year. They are now completed with a degree of taste and magnificence far surpassing the old; and the initiation of the restored institution is to take place this year, at the Jubilee of the great reformation of the church, which was introduced into Denmark in the year 1536. Besides the proper buildings of the university, there are four houses (formerly they were eleven, but seven were burnt in the year 1807,) in which four of the eldest professors with their families reside free of expence. The university also possesses funds derived from considerable legacies and stipends, and which are destined for the support and benefit of the students, for the enlargement of the library, for defraying the travelling expenses of promising candidates, &c.

Amongst these are the following: 1. The *Community* and *Regents ;* the first is a weekly stipend, and the second is a free lodging for one hundred students at the *College of Regents,* a large edifice, founded by King Christian the Fourth. 2. The *foundation of the College of Walkendorff,* the oldest in the records of

Denmark, having been instituted in 1595, by C. Walkendorff, steward of the Empire, for the free residence of sixteen students. 3. *Borch's College*, called also "Collegium Medicum," built and endowed by the learned C. Borch, for the support of sixteen students. 4. *Elersen's College*, founded and endowed in the year 1689, by Jörgen Elersen, counsellor of state, also for the support of sixteen students. Independently of these foundations, many bequests have been granted to the university, the interest of which is appropriated to the support of indigent students and to the general extension of literature.

In order to authorize a student to be matriculated at the university, and to get admittance to the lectures, a public examination must be gone through: this is held once annually (in October,) and is called "examen artium." At this the candidate, recommended by the rector of a preliminary school or by a private teacher, is examined publicly, both by means of written exercises and *viva voce* in every thing, relating to "humaniora," viz. Latin, Greek, history, geography, mathematics, religion, the living languages, &c. The examinations are conducted by the professors in the Philosophical Faculty. It is not allowed to any student to undergo this examination before the age of seventeen; or, at least, a dispensation from this rule can only be granted by a special permission of the *Royal Board of the University*, which will be noticed hereafter. The examination is one of the most impartial and at the same time the most rigorous; and no one who is deficient in the knowledge of the several branches of classical and literary education can obtain a character, (there are three,) which will enable him to procure "testimonium civis academici;" in other words, which will gain him admittance to the university, and confer on him the name of "student;" a name, which in Denmark is more respectable than anywhere else. Matriculation without this "examen artium" is only allowable to those who, after having been matriculated at some other university, intend to continue their studies here or to take the degree of doctor.

As a stimulus to diligence and literary labours, eight prize-essays are annually proposed in eight different branches (theology, law, medicine, philosophy, mathematics, philology, history, and æsthetics), to which only students and candidates without any public office are allowed to aspire. The prize is a golden medal (on one side of which is the figure of Minerva, and the other a wreath of laurel, with the legend—"Studio et Ingenio,") or the sum of 5*l.*

The number of students at the university of Copenhagen has been increasing for many years; and, considering that these consist of natives only, the fact proves a laudable wish of the Danes in general to give their children a literary education. It is even very common for the sons of tradesmen to be brought up at the university. One of the causes which contribute to keep up the number of students is the comparatively small sum of money required for matriculation at the university (sixteen dollars, or 1*l.* 12*s.*) In order to prove the increasing number of students, I subjoin the following table, by which it will be seen that, even after the year 1811, when Norway got its own university by the bounty of the present king, the number did not diminish very much, and has since been increasing considerably.

| Years. | Students. | Years. | Students. | Years. | Students. |
|---|---|---|---|---|---|
| 1808 | 90 | 1813 | 84 | 1818 | 111 |
| 1809 | 83 | 1814 | 107 | 1819 | 102 |
| 1810 | 83 | 1815 | 98 | 1820 | 139 |
| 1811 | 183 | 1816 | 86 | 1821 | 129 |
| 1812 | 102 | 1817 | 116 | 1822 | 153 |

&c. &c. &c. At present the number of students is from 700 to 800.

But whilst there was so considerable a number of students at the university, the proportion of medical students was small, being not more than one or two in the hundred. The chief reasons of this were undoubtedly the greater expenses which medical study involves, and the few public offices in medicine compared with those in law and in theology. But in later years the number of medical students has increased considerably, so that there are now often ten or twelve in every hundred who are matriculated. The natural consequence of this great increase has been an overwhelming number of practitioners, who, however clever and active, are not able to get any public employment, and, consequently, only with difficulty and after long struggles are enabled to earn a livelihood. Nevertheless, they continue to form part of a class the most respectable in morals and the most liberal in principles.

### II. THE MEDICAL FACULTY.

In the medical faculty at the university of Copenhagen there are three ordinary and two extraordinary professors, who are appointed by the Royal Board of the University, according to a recommendation of the Faculty, but which choice must be approved of by the king. They all get an annual salary; and from among the ordinary professors there is every year elected in rotation a Dean, who superintends the affairs at the Faculty. Besides these five professors, there may be an unlimited number of private teachers or lecturers, yet not without having taken previously the degree of doctor at the university; otherwise, a special permission is to be obtained. There are at present only two such private teachers, so that the number of lecturers at the Danish Medical Faculty does not amount to more than seven. This is a number which certainly is not at all adequate to the teaching of the numerous branches of medicine; and, accordingly, several branches, as for instance dietetics, military hygiène, semeiotics, the history of medicine, morbid anatomy, &c. are not at all taught. This is a deficiency in the Faculty, which we hope will soon be remedied by the union of the medical faculty with the other public establishment for medical and surgical education, of which I shall afterwards speak.

The following are the names of the teachers, and the subjects belonging to their respective chairs:

*Ordinary Professors.*

| | |
|---|---|
| J. L. Saxtorph, . | Surgery, Midwifery, the Diseases of Women and Children. |
| D. J. Herholdt,* . | Human Physiology. |
| C. L. Bang, . . | Pathology, Therapeutics, Clinical Medicine. |

*Extraordinary Professors.*

| | |
|---|---|
| D. F. Eschricht, . | Anatomy and Human and Comparative Physiology. |
| C. Otto, . . . | Pharmacology and Forensic Medicine. |

*Private Lecturers.*

| | |
|---|---|
| E. Svitzer, . . . | Anatomy and Dissections. |
| S. T. A. W. Stein, | Anatomy. |

In the Philosophical Faculty of the university are given lectures on the collateral branches of medicine, botany, chemistry, zoology, mineralogy, &c.

It is of course here, as in other universities, that the professors are not restricted to give lectures only in those branches to which they are appointed, but may lecture upon any other they choose. This, nevertheless, very seldom happens, and it will at once be seen on what unequal terms the different branches of medicine stand at the Danish university. Anatomy has three lecturers, physiology, two; while semeiotics, morbid anatomy, mental diseases, &c. have none at all. The same is the case with some other branches of therapeutics, as for instance, diseases of the eyes, of the ears, of the teeth, &c.; an anomaly which also will find a remedy in the above-mentioned union of the two establishments for medical education. Another defect in the Medical Faculty at the university is the very small salary which the five professors receive for their labours: although they lecture almost every day during eight months of the year, the ordinary professors do not receive annually more than 1000 Danish dollars (100*l.*) and the extraordinary ones only 800 (80*l.*) The natural consequence of this is, that the medical professors, as they do not get one farthing from the students,

* Since dead.

the lectures being all public and gratuitous, in order to obtain a livelihood, are obliged to endeavour to procure some additional office, and also to enter into private practice; the consequence of which is the impossibility of their paying that exclusive attention to their professorships which these require. Yet it is exactly on this false principle, that the medical professors will be able to increase their income by practice, that the Royal Board of the university is acting in appointing a less salary to them for their labours at the university than to the professors of the other faculties; a principle which certainly is to be blamed in the severest terms, inasmuch as the salary should be sufficient to enable the professors to do without practice or any other offices, so as to devote themselves entirely to the duties of their chairs. The professors may certainly, besides their public lectures, give private ones, i. e. such as the students must pay for; the sum paid for such private lectures is only five dollars (2*s.* 6*d.*) for each [course?] and even this small sum the medical student is so unaccustomed and so disinclined to pay, that he very rarely attends such lectures. And truly, for this reason, the professor is even ashamed to ask his fee of the few who do attend. The same principle operates also as a cause of the impossibility of the professors occupying themselves with literary labours: for who that is forced to spend the whole day in the labours of an active practice is able to devote the few remaining leisure hours to literary studies? or who that has two offices to attend to (and all the professors of the Medical Faculty have more than one office,) can be expected to have much time left for any scientific occupations, except such as are involved in the discharge of the duties belonging to those offices?

Before he can undergo his examination at the Medical Faculty of the University, the medical student must have gone through the following preliminary trials:—He must be matriculated as student at the university, i. e. he must have taken his examination at the "examen artium." 2. He must furthermore have undergone the *philosophical examination* (examen philosophicum), which commonly takes place in the course of a year after the "examen artium," and is divided into two parts, at the first of which (examen philologicum) the candidate is again examined in Greek, Latin, history, mathematics, archæology, and natural history; and at the second (examen philosophicum proprie sic dictum,) in physics, astronomy, and the several branches of philosophy (logic, morals, metaphysics, &c.) The object of these preliminary examinations is to give the examiners a proof of the progress of the student in his literary career, and to oblige him to imbibe a knowledge of the natural sciences, without which he never will become a true, learned, and scientific physician. After these two examinations, the student may apply himself to the study of medicine; and when he requests to be examined at the "examen medicum," he must, 3, in addition to the foregoing, produce certificates of having attended the several lectures of the Professors of the Faculty, and of having visited the hospitals.

There is no period fixed for the study of medicine, as at the Austrian and many other universities; neither are certain lectures appointed to be attended the first, the second, the third year, and so on. Such supererogatory interference certainly only tends to stifle genius, and to make diligence and industry of less avail than they ought to be. It is left entirely to the candidate in how many or in how few years he shall finish his studies, but the usual term is four or five years.

When the candidate is admitted to the "*examen medicum rigorosum*" by the Medical Faculty, he must undergo the following trials of his knowledge:—1. He must examine a patient, newly taken in at the Fredrik's-Hospital (the first in town), explain the symptoms of the case, assign it a name, tell the seat of the disease and its remedies, prescribe the indicated medicine and the diet, &c. 2. He must give, in Latin, written answers to a physiological and a therapeutical question. 3. He must make two anatomical dissections in the dissecting room. 4. He must perform two surgical operations, explain their nature, the different modes of proceeding, the possible consequences, &c. 5. Finally, he is examined

*viva voce* in all the different branches of medicine by all the Professors of the Faculty. The written answers to the questions are drawn up in the presence of an inspector, and no other help but a Latin dictionary is allowed. All the other examinations are public, and they have been hitherto in the Latin language, the explanations of the surgical operations excepted. The oral examination lasts a whole day for each candidate, every professor examining during the space of an hour or an hour and a half.

When the examination of the day is finished, the candidate and all the auditors leave the room, and the professors hold a council, in which they determine by vote the general character which is to be given to the candidate. There are three such general characters: *Laudabilis, haud illaudabilis,* and *Non contemnendus.* The last named is seldom given, the candidate entitled to no other being commonly rejected; but it is proper to state, that the examination of the Medical Faculty is conducted in the most liberal spirit, and that greater notice is taken of the general knowledge and ability of the candidate than of refined subtleties and smaller deficiencies. When the professors have agreed upon the general character, the candidate is called in, acquainted with the result, and an oath in the following terms is taken from him: "Gratulamur tibi de exhibito eruditionis specimine et speramus te eandem optimo modo in emolumentum societatis et proximi adhibiturum atque magis magisque aucturum nec non leges, quæ medicum et chirurgum obstringunt, tibi cognitas redditurum et impleturum, in cujus promissi sanctam observantiam te oportet dextrum porrigere." The testimony of having undergone the "examen medicum" is this: "anno [ ] die [ ] speciminibus scriptis et tentaminibus scriptis rite absolutis N. N. philosophiæ et philologiæ in alma universitate Havniensi Candidatus et Medicinæ Studiosus examini medico chirurgico practico rigoroso se subjecit indeque (*Laudabilis* or *Haud illaudabilis,*) discessit."

The sum which the candidate has to pay for admission to the examination is very small, not amounting to more than twenty dollars (2*l.*), of which the Dean receives five, the eldest professor five, and the youngest (as Secretary of the Faculty,) two dollars. Examinations take place twice in the year, in the spring and in the autumn.

When the candidate has gone through the *examen*, he is immediately allowed to practise medicine and surgery wherever he pleases in the Danish States, the degree of doctor not being necessary to practise. The degree of M.D. is in Denmark but a literary honour, which only is required when any one aspires to a professorship at the university. It will from this at once be seen, how much the arrangements differ in this respect from those of the universities in Germany, and particularly in Prussia. If a foreigner wishes to practise as physician or surgeon in the Danish States, he must, besides being previously matriculated at another university, undergo a preliminary examination in the same branches of classical education that are required at the "examen artium" and "examen philosophicum," and not until then is he admitted to the "examen medicum rigorosum."

### III. THE DEGREE OF DOCTOR OF MEDICINE.

Whoever wishes to take the degree of M.D. at the University of Copenhagen, must necessarily, 1. be matriculated at the university and have taken the "examen philosophicum;" 2. submitted to the "examen medicum" and have obtained the first character (laudabilis); and 3. must be "Licentiate of Medicine." In order to become licentiate, he must write a medical dissertation in the Latin language, and deliver it to the Medical Faculty to be examined; when it is admitted to be well and scientifically written, the candidate must get it printed, together with the sentence passed upon it by the Faculty, and then defend it publicly, in the Latin language, in the hall of the University, against two professors of the Faculty appointed for this purpose, ("opponentes ordinarii,") and against any one of the auditory who may choose to question it. If the candidate defends his dissertation in an able manner, the Faculty announces it to the Royal Board of the University, which, with the King's permission, assigns the name of "Licentiatus Medicinæ" to him. Only those who are in public offices, or have acquired a celebrated name, are allowed to take the degree of doctor before being "Licentiati;" but, if that is the case, the degree of doctor is obtained by writing once more a Latin dissertation, and doing all the above-mentioned things over again, defending the essay publicly, &c.

It is to be remarked that much stress is laid upon the taking of the degree of licentiate and doctor, and that nobody is allowed to receive them without having written a really good essay, and given proofs of solid knowledge and a true scientific spirit in the composition and defence of it. The name of a Danish Doctor of Medicine is consequently held in much greater respect than that of a German; and many of the candidates have by their inaugural essays really contributed to the progress of science. At the public defence of the dissertations, which usually lasts a whole day, the candidate is allowed an assistant ("Respondens," as he is called), who commonly is younger than the candidate, and whose name appears on the title-page of the printed dissertation.

The sum which is paid for admittance to the degrees of licentiate and doctor is a small one; being for the first twenty-five dollars (2*l.* 10*s.*); for the second, seventy dollars (7*l.*) of which the Dean at the Faculty receives forty dollars (4*l.*) The greatest expenses are incurred in the printing of the dissertations; about one hundred copies of which are distributed gratuitously to all the professors of the university, to the public libraries, &c.

The degree of doctor is held in such high esteem, that a special academical meeting is appointed for the occasion, and previously to the ceremony a program is issued, written by the Professor of the Latin language at the university, and accompanied by a note of the life of the candidate. At the inauguration, the Dean of the Medical Faculty ascends the chair, and, after a Latin speech, composed for the occasion, upon one or other fitting medical subject, he confers the degree, making use of the old symbols: the *book,* the *hât,* and the *kiss.* After this, the new-made doctor ascends the rostrum, and in a Latin speech returns thanks for the honour conferred on him.

Every one bearing the degree of doctor is entitled as well to aspire to a professorship as to give public or private lectures; and, if he makes application for a public appointment, he is, *cæteris paribus,* preferred to other candidates. He has also a certain rank in the state, for which however he must pay a certain annual tax. These are all the advantages of being a doctor of medicine in Denmark. The real value of the degree is only known to literary persons acquainted with the signification of the word and the thing; for it is the custom in Denmark, or elsewhere, to call every practitioner "doctor." As has been already stated, no one can get appointed to any of the public or royal medical offices in Denmark except those who have undergone the examination of the Medical Faculty at the university, although the degree of doctor is not required. But these offices are few in number, not amounting in all to more than twenty-three. They are as follows: five professors at the university; eleven county physicians; one physician of the Public Lunatic Asylum; two physicians of the court, (whose duty comprehends attendance on all the inferior officers and servants;) one physician of the poor in Aalberg in Jutland; one county physician in Iceland; one in the West Indies; one physician of the King. The prosector at the university, and the physician at the second hospital (the general) in Copenhagen, have both other surgical offices. The professor of midwifery and surgery at the university, J. S. Saxtorph, is also physician of the lying-in hospital; the professor of physiology, J. D. Herholdt, is the first surgeon of the navy; the professor of therapeutics, C. Bang, is physician of the first hospital (Fredrik's) in Copenhagen; the professor of anatomy, F. D. Eschricht, is physician of the Foundling-Hospital; and the Professor of Pharmacology and Forensic Medicine, the writer of this, is physician of the public Penitentiary. The first and the last offices are not always united with the professorships, but the others are always so. Of medical officers not royal there are three physicians of reserve and eight assistants (called candidates) at the hospital.

### IV. SCIENTIFIC ESTABLISHMENTS.

1. *The Dissecting Room and the Anatomico-Pathological Museum.* The last mentioned is a very valuable collection of anatomical, pathological, and zootomical preparations, and contains about 1400 species. For the support of this establishment, 10,000 dollars (1000*l.*) are set apart, which are a portion of 294,000 dollars (29,000*l.*), that were left as a legacy to public establishments by a rich Jewish merchant, Amsel Meyer. Another Anatomico-pathological Museum is at the College of Surgeons; and a third at the Veterinary School.

2. *The Royal Museum of Natural History.* This was founded in the year 1796, by the union of several minor museums, and divided into two classes—zoology and mineralogy. The first consists of a collection of European birds, presented to the museum by Captain Wöldicke; a collection of African birds, presented by Mr. Hancke, Danish consul at the Cape of Good Hope; of birds from South America, &c.; and a collection of insects from the most remote period, described by the celebrated Fabricius in his work on insects, fishes, reptiles, &c. The second collection (of minerals) is extremely valuable: the minerals are from Iceland, Feroe, Greenland, Hungary, Austria, England, &c. The museum is open to the public twice in the week.

3. *The Museum of Natural History at the University.* This contains many zoophytes, insects, birds, &c., but more particularly minerals, which are used for the illustration of the lectures on mineralogy.

Besides these public museums of natural history, there are several private ones; as, for instance, one that belongs to the hereditary Prince Christian Frederik; one belonging to the apothecary Becker, &c.; but what deserves to be particularly mentioned here is a large collection of all sorts of animals belonging to a private "*Society for Promoting the Knowledge of Natural History,*" which has been founded only three years, but consists of a very great number of members. The museum is open every Sunday to the members of the society and their families; and once in the year, during a fortnight, to the public. On every other Sunday in the winter a popular lecture is given alternately by Professor Eschricht at the university; and Schouw, professor of botany, upon some subject in natural history.

Another private society, which also has been founded lately, is that "*for the Promotion of the Natural Sciences,*" which owes its origin to the celebrated Oersted, the greatest ornament of the university. This society, which has done much for the natural sciences in Denmark, has an excellent museum and maintains lecturers on the natural sciences in all the Danish provinces. Every other Sunday in winter popular lectures are given to a large audience of members by Oersted or by Forchhammer, professor of mineralogy at the university, and a celebrated name in chemistry.

The writer of this Report possesses a good Phrenological Museum, consisting of natural skulls and casts, the last for the most part from Edinburgh.

4. *The Collection of Philosophical Instruments.* This belongs to the university, and is very rich and valuable: it is under the superintendence of Oersted. The veterinary college has also a small collection of instruments.

5. *The Botanical Garden.* This was founded in the year 1778, and differs in this respect from most other botanical gardens that it is situated in the middle of the town behind the palace of Charlottenborg, (the royal academy of arts.) Although it belongs to the university, it is principally supported by the king; and is undoubtedly one of the most extensive botanical gardens of Europe. The number of plants amounts to 9000, and from four to five thousand different seeds are annually sown. The garden is daily open to every student from eight to twelve, and from two to seven o'clock. To the garden belongs an excellent library, consisting of a rich store of botanical books, plants, herbaria, &c. The manuscripts, and a Herbarium of more than 20,000 species of the late celebrated

Vahl, are the treasures of the establishment. The Professor of Botany at the university is inspector of the garden: the present is the celebrated T. W. Horneman, who has published a complete catalogue of the plants of the garden, accompanied with a botanical description, entitled, "Hortus regius Botanicus Hafniensis," in two volumes, and also a very valuable systematical description of all the plants of Denmark ("Plantelære," 1821.) The celebrated botanical work with plates, entitled "Flora Danica," also continues to be published under his inspection.

6. *Public Libraries.* Of these there are several; the following are the principal:

*a.* The *Royal Library.* This was founded by King Frederick the Third, in 1665, and is supposed to be one of the first royal libraries in Europe, containing about 400,000 volumes. It consists of nine magnificent rooms. The largest of them is 260 feet in length, and contains every work of antiquity; around it is a beautiful gallery appropriated for the same use. An isolated part of it is the *Northern Library,* which has been arranged since the year 1781, and contains the whole literature of the Danish States, and very much of the Swedish. Few public libraries have a more extensive collection of manuscripts, atlases, and engravings. The engravings are from the most ancient period, and are divided into three classes. The eldest collection consists of 47,228 engravings, bound in fifty-five large volumes, which were collected in the seventeenth century, under the reign of King Christian the Fifth, and are of the greatest antiquity. A later collection, formerly belonging to Mr. Wasserschleben, Privy Counsellor to his Majesty, consists of 29,016 engravings, and is bound in 212 large volumes. According to a law passed in 1821, two copies of every book printed in the Danish States are to be delivered to the library. It is open every work-day from eleven to one o'clock, and in the reading room every one may, during these hours, read and make extracts from books; and they are also most liberally lent to any one who either is known, or procures a written security from some one who is so.

*b. The Library of the University.* This is arranged in a spacious saloon over Trinity Church, and contains about 100,000 volumes. The manuscripts, presented to this library by Arne Magnussen, are also very numerous, and illustrate the antiquity, geography, history, and language of the northern nations. Independently of this extensive bequest of books, he left to the university a considerable legacy, the interest of which is appropriated to the extension of literature. The library is also much indebted to Rasts, the late celebrated linguist, and to Dr. Wallich, at Calcutta, for a fine collection of Oriental works, which are here deposited; it is indebted to the late Count Moltke, Minister of State, for a collection of works in natural history; and to the Counsellor of State, J. H. Schou, for a large collection of books belonging to the science of law. The voluminous manuscripts of Iceland are here deposited, and are very remarkable; but the greatest curiosity is one volume in Runic characters, which is the only book of this description in existence; it contains the most ancient laws of Denmark. The old seals and letters of Norway also deserve to be mentioned, and an ancient copy of the celebrated Edda. To this library also a copy of all Danish books must be delivered gratuitously. A reading-room is attached to the library, and is open daily, from ten to two o'clock; books are also lent from this library.

*c. The Classenian Library.* This consists of 30,000 volumes, and possesses, in particular, books in mathematics, natural history, economy, geography, travels, &c. Major-general J. F. Classen, and his brother P. H. Classen, privy-counsellor to his Danish Majesty, were founders of this library. Amongst the valuable collection of books is a superb edition of botanical paintings in seventy volumes, by J. T. Herner, and which was presented by the present king, when prince-royal of Denmark. The library has a reading-room, and is open to the public four days in the week, from eleven to two o'clock, and books are lent, as in the other libraries.

*d. The Classenian Library for the use of Medical Men.* This consists only of medical books, which already amount to four thousand, and are increasing considerably every year. It is supported by a bequest of the above-mentioned brothers Classen, and by pecuniary contributions from the members of "*the Classenian Society of Literature for Medical Men*;" among whom the books and journals that are bought circulate, before they are deposited in the library. It is open to all medical men one day in the week, from eleven to one, and books and journals are lent.

Besides these libraries, there is one at the Veterinary School, one at the Royal College of Surgeons, &c., besides many large private ones.

B. THE ROYAL COLLEGE OF SURGEONS, OR THE SURGICAL ACADEMY.

Surgery has, since the year 1736, had its own school in Copenhagen. It formerly was called "Theatrum Anatomico-Chirurgicum;" but, in the year 1785, the institution was considerably enlarged, and got the above name, together with a new magnificent building, which was opened in 1788. This college, more particularly in the beginning, has contributed much to the progress of surgery in Denmark. Before its institution, surgeons and barbers were one and the same thing, and, of course, no well-bred young man or of the more respectable classes devoted himself to surgery.

All surgical offices, military and civil, are now only filled with gentlemen who have studied and passed their examination at this college.

The college consists of three ordinary professors, one adjunct, two lecturers (one in chemistry, another in anatomy,) four teachers, (who are called "surgeons of reserve," and are always young men, very often such as have been examined not more than two or three years before at the college,) and one candidate teacher.

The following are the names of the lecturers, and the departments taught by them respectively:

*Ordinary Professors.*

C. Fenger, (the surgeon of the king,) Surgery and Midwifery.
C. Withusen, (now general-director of the college,) Anatomy and Diseases of the Eyes.
A. Callisen, . . . . . . Surgical Pathology and Therapeutics.

*Adjunct Professor.*

J. C. J. H. Gundelach-Moller, Forensic Medicine and Clinical Surgery.

*The Lecturers.*

G. Scharling, . . . . . Chemistry.
C. L. F. Henck, . . . . Anatomy.

*Surgeons of Reserve.*

M. Diörup, . . . . . . Materia Medica.
J. F. C. E. Starck, . . . Botany.
J. Rorby, . . . . . . Special branches of Surgery and Pathology.
J. Pape, . . . . . . . Special branches of Anatomy, and Therapeutics.

*Candidate Teacher.*

J. E. Larsen . . . . . Special branches of Surgery.

All the lectures are public; no private ones are allowed.

It will be seen that there is abundant means provided for teaching anatomy at the college, as well as at the Medical Faculty of the University. Indeed, Copenhagen is one of the places where this branch of medical science may be studied with the greatest advantage, not only on account of the able teachers here, but also because the student has the greatest facility and best opportunity of dissecting, both at the Faculty and at the College. In both, the dissecting rooms are open every work-day, and dead bodies are to be got at an extremely small cost. From the hospitals the dead bodies of poor persons, who either die without any relations or with relations that do not care for them, are often to be had for no more than two English shillings. The standard price at the Faculty for a body is four shillings. Both institutions are, according to law, alternately and gratuitously furnished with the dead bodies of the criminals who die in the different public penitentiaries.

The same defect already noticed respecting the lectures at the Medical Faculty is also observable at the College. There are no lectures given on physiology, dietetics, morbid anatomy, on the diseases of the teeth, of the ears, of the mind, &c.; and very often the most important branches of surgical and medical knowledge, as, for instance, semeiotics, are left to the junior teachers, who of course want the necessary practical experience; faults which, as already mentioned, would be remedied, if the College of Surgeons was united with the Medical Faculty.

In order to show how small the remuneration is which in Denmark is given for scientific labours, I must here also notice the salaries of the lecturers at the College. The three professors get no more than 660 dollars (60*l.*) annually; the adjunct professor fifty dollars (5*l.*;) the lecturer in chemistry four hundred dollars (40*l.*;) the four surgeons of reserve, 144 dollars (14*l.*)

To the College belong, 1. a very good *Anatomical and Pathological Museum,* which has an inspector, and is open to the student twice in the week in the summer, from twelve to two o'clock, at which time the inspector, Mr. Ibsen, explains the preparations; 2. a *Library of Surgical and Medical Books*, of which Professor Callisen is librarian; and, 3. a *Collection of Surgical Instruments*, which, however, is as yet very small.

To enable one to study and to be examined at the College of Surgeons, it is not necessary to be matriculated as "civis academicus" at the university, but the lectures are open to every one who pleases to attend them; and, when anybody wishes to be examined at the College, he petitions the professors, and delivers at the same time his *biography*, which indicates his native place, his age, parents, the school he has attended, his literary and medical education; and this must, of course, be accompanied by testimonials of having attended the lectures at the College, the hospital, and the Clinics.

The candidate, when admitted, must either present to the College an anatomical preparation made by his own hand, or, instead of this, pay ten dollars, (1*l.*;) so that either nothing is paid in money or only this small sum. He must, in the first place, give written answers to a surgical, therapeutical, or medico forensic question, in the Danish language; in the second place, he must make an anatomical dissection, (which commonly requires a whole day;) thirdly, he must examine a patient in the surgical clinic in Fredrick's Hospital; and, finally, he is examined, *viva voce*, in the Danish language, by the professors, the adjunct, and the lecturer in chemistry, during eight hours, which are distributed over three days. At the end of the examination on the third day, the candidate is called in, and is made acquainted with the particular character he has received; and, on being admitted, the following oath, in Danish, is taken by him: "After having publicly proved my medical and surgical knowledge, I do hereby promise and engage myself, with oath and hand, to the Board of the College of Surgeons, that I shall always endeavour, in my function as practitioner, to employ my art zealously for the benefit of the community and my fellow-creatures; that I will take equally scrupulous care of the poor and the opulent, without any personal regard; strive to enlarge my knowledge, and learn and comply with the laws and ordinances that belong to my profession."

There are six general *characters* given at the examination, a number which certainly is too great, although in fact they are reduced to three principal ones, as every character (even the lowest,) entitles the candidate to practise medicine as well as surgery everywhere in the Danish states; but, of public offices, they can only get such as belong to the surgical department: yet these surgical offices surpass in number by far the medical ones. The royal public surgical offices amount to sixty-two military and eighty-four civil; but, of surgical offices that are not royal and very subaltern, there are ninety-four in the army and seven in the navy; besides an office for a surgeon of reserve at the second hospital in town, (the General Hospital,) for one at the Military High School, and offices for eight assistants ("surgical candidates") at the two hospitals. Nobody, who has only been examined at the College of Surgeons, is allowed to take the degree of doctor.

The number of students at the College was formerly from 130 to 150, but this number has also increased in later years, (now it amounts to 200, fifty of which number being without any academical education;) and, when it is considered that the surgical offices in Denmark are only filled by those who have been examined at the College; that these offices far surpass the medical ones in number; that the two examinations at the university (the "examen artium" and "examen philosophicum,") are not required at the College; that, furthermore, nothing, or (failing an anatomical preparation) only ten dollars (1*l.*), are paid by the surgical candidates, it certainly is not to be wondered at that many more get examined at the College than at the Medical Faculty, and that even the greater part of those who submit themselves to the examination of the Faculty previously go through the examination at the College, in order to be entitled to make application for surgical offices. The folly of having two institutions where the same branches of the art are taught, the same examinations made, (differing only so far that the Latin language is used at the one, the Danish at the other,) is evident; and, were it only on this account, a reformation of the whole medical and surgical instruction in Denmark is loudly called for.

### C. THE MEDICAL PROFESSION IN DENMARK.

From what has been stated of the qualifications requisite for admission to the examinations at the Medical Faculty at the University, and at the College of Surgeons, and of the extent of knowledge required at both in all the different branches of medicine and surgery, it is evident that the state has done every thing in its power to secure the aid of able and enlightened men in an art upon which depend the health and life of its subjects. Every physician and surgeon in Denmark gets an education which qualifies him to maintain the dignity of his profession, as a worthy member of a class that is generally considered as one of the most respectable and most liberal. The Danish medical men are usually held in high esteem. We have seen how much the doctor of medicine deserves respect from his scientific acquirements; and Danish physicians and surgeons are so honoured abroad, that very often Swedes come to Copenhagen in order to be treated by them. Mountebanks and quacks amongst the Danish medical men are likewise rare. This is the more to be wondered at, since the number of both physicians and surgeons is increasing greatly every year, in every part of the Danish dominions. In Copenhagen alone, with a population of 110,000, the number amounts already to 183, and that the condition, as well of those endowed with public offices as of mere private practitioners, is none of the most brilliant, will be evident from the following facts.

The salary which the state gives to those in royal public medical or surgical offices is extremely small: to the surgical, 150, 200, 400 dollars, (15*l.*, 20*l.*, 40*l.*;) to the medical, 400, 600, 900 dollars, (40*l.*, 60*l.*, 90*l.*) according to the time of service. In order to gain a livelihood for his wife and children, the officer must devote all the hours of the day to practice, which in the country is burthensome in the highest degree; the county in which his office is, being often forty English miles in circumference, and every year younger colleagues are settling in his county, who take more and more of his practice from him. Still worse is the condition of the mere practitioner without any public office: he is not, as in England, paid for every visit to the patient, but gets his fee after his recovery; or, still more commonly, he attends families as "house-physician," paying visits every time a member of the family gets sick, and receiving his fee every new-year's day. This fee for the attendance of a whole year amounts usually to from twenty-five to thirty dollars (3*l.*); and this even from families where there are many children, and where, upon the whole, perhaps only one month of the year has been without attendance. Only very few give fifty dollars (5*l.*); and he is a *rara avis*, and falling only to the lot of the older and very celebrated physicians, who pays the exceedingly large fee of 100 dollars (12*l.*) on new-year's day! For the care of a patient who is not attended by the medical men in quality of house-physician, he must think himself well paid when he gets ten or fifteen dollars (1*l.* or 1*l.* 10*s.*), and this perhaps after an attendance of several months. Many, even of the higher classes, pay nothing at all, and on that account, like good politicians as they are, change their physician every year or every time they become sick.

There is certainly a legal charge fixed for every visit the medical man makes, and for every receipt he writes; but such is the custom of the country, that a physician dare never ask his due, for by so doing he would be accused of sordid avarice, and be obliged to yield his patient to one or other of his many brethren, and at last lose entirely what practice he had. Only in the country the medical men who are not paid are accustomed to give in their bills, but many of their patients bitterly complain of such a mode of proceeding; and yet the charge for travelling fifteen English miles, and in this manner employing at least eight hours, is no more than five dollars (half a crown!) The Danish physician is really, to say truth, most fortunate in this respect when one of his patients, particularly a wealthy one, *dies!* for he is then always *requested* to give in his bill for his attendance; and he may then charge the heirs with a sufficient sum without risking objections, although this now and then happens, and he is, according to law, the first paid of all the creditors.

The miserable and unjust condition of the Danish medical men in all these respects need not be pointed out. It should not be left entirely to the arbitrement of patients to pay what they please: it should be a strict legal order for the medical man to deliver his bill according to a legal charge; it would not then, as now, be thought strange and self-interested to do so; the wealthier and more grateful patients might still, when so minded, give *more* than the legal demand; and, from so liberal a profession as the medical, the poor certainly would not have anything to fear by such an arrangement; for who would charge them? If, according to the present system, medical men, poor themselves, are sometimes obliged to charge or receive a fee from them, would this not happen much more seldom when they received a better and sure remuneration from the more opulent? As it is, the young medical man cannot gain a livelihood by practising, and the elder ones must, by the *number* of patients, try to compensate for the smallness of the fees: and this is one of the reasons why the most celebrated and most able Danish practitioners, endowed with the most valuable experience, are unable to do anything towards the advancement of the science.

In order to lessen the probable want that may arise to the family of a medical man who dies in poverty, there exist two *benevolent medical societies* for the relief and support of the widows and children of deceased medical men; one in Copenhagen, another in the provinces.

An amiable trait in the character of the medical profession in Denmark is the harmony that exists between its members. It scarcely ever happens that one practitioner, from sordid motives, blames the conduct of the other at the sick bed: one never interferes with the practice of another; and, if one is called to a patient whom he supposes to be attended at the time by another, or who has his own house-physician, he never prescribes for the patient without previously having ascertained whether he is called according to the wish and the consent of his colleague, and without having consulted with him: if he is called contrary to the consent of his colleague, or without his knowledge, he refuses to prescribe at all. On the other hand, in dangerous or obstinate cases, it is very common for one physician to take another with him in consultation; and there is not one single instance on record where such consultations have not ended in the most friendly manner.

The harmony and the scientific intercourse amongst the medical men in the Danish capital are supported by two *medical societies*.

1. The *Royal Medical Society.* This was founded as early as the year 1772, and counts now amongst its members all the elder and younger literary and able physicians and surgeons in Denmark. It consists of honorary, native, ordinary, and foreign corresponding members. The present number of the native ordinary members is forty-five; amongst the foreign members (sixty-two) are the following Englishmen: Mr. G. Wilkinson, of Sunderland; Sir Astley Cooper, of London; Dr. John Thomson and Dr. Wishart, of Edinburgh; Mr. Wardrop, Mr. Howship, and Dr. Elliotson, of London. The society meets only in the winter season, and on every other Thursday, at seven o'clock. At the meetings, original essays, remarkable cases, &c., are read by one or more members, and medical matters, and particularly epidemic diseases, are discussed, &c. The most scientific and friendly spirit pervades these meetings, and, although now more than sixty years have passed over the society, its zeal for the science continues unabated. It has special laws, which have lately been revised. The society has hitherto published seven volumes of its Transactions, ("Acta Societatis Regiæ Medicæ Hafniensis,") but the greater part of later transactions have been published in the Danish medical journal, "Bibliothek for Læger," (the Physician's Library,) and (the custom of writing in Latin having ceased,) will in future continue to be so.

2. The *Philiatria.* This is a medical society of younger date, having existed only four years: it consists only of the younger members of the profession in Copenhagen. It meets every Tuesday in the winter, at six o'clock; and its object is rather to discuss medical matters than to read essays, although this sometimes happens. It consists at present of thirty members.

From the existence and flourishing state of two such institutions in a town of only third rank, it may be inferred that a great love for science and literary pursuits exists in Denmark; and certainly the Danish medical man is thoroughly acquainted with the progress of medicine and the medical literature of all foreign countries. In this respect he does not yield to the medical men of any other country; and, from his necessary study of foreign languages, which are all known and spoken by well-educated Danes of the better classes, he even surpasses, in this particular, as well the English as the French and the Italians. In fact, medicine is the branch of science which has reached the highest degree of perfection in Denmark. The King's Library has a rich stock of medical works and takes in many foreign medical publications; the Medical Library, formerly noticed, is still richer in medicine, and subscribes to almost all the medical journals of Europe, the Italian alone excepted, which cannot be had without great difficulty. In the provinces also there are circulating medical libraries; and many medical men have good private libraries, and likewise subscribe to journals.

Notwithstanding this familiarity with medicine and its literature abroad, the medical men of Denmark are not prone to adopt new methods or new systems of medicine; and all the romantic and mystic hypotheses of Germany have been received with distrust, and have not been able to take root amongst us. We have never been either the first to adopt the new before it was sufficiently tried, nor the last to leave the old when its usefulness was proved beyond all question.

In their general methods of cure, the Danish medical men do not commonly use, but rather shun, heroic remedies: as true sons of Hippocrates, they follow his maxims in studying nature, and in endeavouring, in all their treatment, to obey it, and to sustain the *vires medicatrices,* not disturbing them by too active medicine. That, nevertheless, no extravagance in this respect is allowed to take place is proved by a golden book, by the late celebrated practitioner, Dr. Ludvig Bang, entitled "Praxis Medica," written in Latin, and once a standard of medical practice in Denmark. Nor is Denmark a home for quackery, being by far much less filled with quacks than England, France, or Germany. The rights of authorised medical men are strictly secured by laws which prohibit every species of quackery and unauthorised dispensation of medicine. In order to preserve the enforcement of these laws in all their vigour, all physicians and surgeons are bound to put their name and official function on every one of their written prescriptions; and an alphabetical list of all authorised medical men and their assistants is annually distributed among the apothecaries, chemists, and druggists, who are prohibited by a heavy fine from delivering medicine to any other but those who present a prescription signed by the name of a legalised medical man; and it is incumbent upon every assistant (amanuensis) of a practitioner to take his examination within two or three years from the time of his appointment as assistant. All the provincial medical officers and the state-physician in every town must, according to law, report every year to the highest medical authority in Denmark whether any quackery has made its appearance during the year at their place of residence.

This "highest medical authority" in Denmark is the Royal Board of Health, which resides in Copenhagen, and was instituted in the year 1803. According to the royal decree, it now always must consist of ten medical members, five physicians, five surgeons, and two apothecaries. Every thing relating to medicine, surgery, and pharmacy in Denmark, medical police, medical and surgical practice, the state of the apothecaries' shops, the behaviour of midwives, &c., are under the superintendence of this Board of Health. Reports of epidemic diseases, of the state of health of the respective places, of remarkable cases, of unhappy accidents, of the quality of medicines that are sold by the apothecaries, of unauthorised practitioners, &c., must annually be sent to this College of Health by the several county physicians, who again are bound to obtain like special reports from the county surgeons and the mere private practitioners in their counties. The College of Health decides also upon the doubtful mental state of criminals or the accusal, and has likewise the privilege of making proposals to the king about the filling of vacant medical and surgical offices, on which account all applications for such vacant offices are first sent to it; and so great is the impartiality of the king, that he scarcely ever omits to ask the opinion of the College of Health, or acts contrary to it by listening to patrons or friends of the candidates for an office. It is but justice alone to state that the College acts with the greatest impartiality, weighs the respective merits of the many candidates in doubtful cases, leaving the final decision to the king. The only objection that might be made in this respect is that too much stress is laid upon the *age* of the candidates for such offices. The College of Health is also bound, in conjunction with the *Town* physician, to visit the shops of the apothecaries once a year (in the autumn), in order to examine the quality and goodness of all the medicines on sale; on which occasion also many chemical analyses are made.

The principal administration of the functions of the College of Health is undertaken by a Dean, who is annually changed, and by turns chosen among the medical and surgical members of the College. The College assembles once a fortnight (every other Saturday at six o'clock,) in order to examine and decide on the cases that have been laid before it; and these, in the interim, circulate among the members, all of whom, with the dean at the head, are bound to vote upon them.

It is remarkable that the members of this College, although really working hard in the fulfilling of their duties, and although having annually about six hundred different cases to decide upon, do not enjoy any pecuniary emolument or salary for their labours; the dean alone receiving, the year he is in office, 400 dollars (40*l.*), and his secretary 200 dollars (20*l*). It is also a defect in the organization of the College of Health that it cannot refer directly to the king, but that all its decisions and proposals go through the royal Chancery; an arrangement that necessarily must damp the activity and the influence of the College; the Chancery, although not possessing one single man acquainted with medical affairs, often deciding contrary to the opinion of the College.

*Copenhagen; March* 17, 1836. C. O.

